# Evaluation of *Yucca schidigera* extract as feed additive on performance of broiler chicks in winter season

**DOI:** 10.14202/vetworld.2015.556-560

**Published:** 2015-04-29

**Authors:** Sarada Prasanna Sahoo, Daljeet Kaur, A. P. S. Sethi, A. Sharma, M. Chandra

**Affiliations:** 1 Division of Livestock Production Management, Indian Veterinary Research Institute, Izatnagar, Bareilly - 243 122, Uttar Pradesh, India; 2 Department of Livestock Production Management, Guru Angad Dev Veterinary and Animal Sciences University, Ludhiana, Punjab, India; 3 Department of Animal Nutrition, Guru Angad Dev Veterinary and Animal Sciences University, Ludhiana, Punjab, India; 4 Department of Veterinary Microbiology, Guru Angad Dev Veterinary and Animal Sciences University, Ludhiana, Punjab, India

**Keywords:** broiler, feed additives, winter season, *Yucca schidigera*

## Abstract

**Aim:**

*Yucca schidigera* extract has been successfully used as feed additives in the poultry industry. It enhances the growth and productivity in broiler production. Hence, the present study was designed to analyze the effect of *Y. schidigera* extract in growth, carcass quality and behavior along with its economical utility in broiler rearing.

**Materials and Methods:**

Total, 120 numbers of day-old broiler chicks of equal sex ratio were randomly divided into Yucca supplemented treatment and control group, each having 60 birds in three replications of 20 numbers. The feeding management and rearing conditions were similar for all the groups as per the standard except the Yucca supplementation in the treatment group @ 125 mg/kg of feed. The parameters with respect to growth, carcass, behavior, and litter content were recorded as per standard procedures.

**Results:**

The Yucca supplementation can effectively enhance growth of 173 g in 6^th^ week by utilizing lesser feed intake than control group, which ultimately proves better feed conversion rate, protein efficiency ratio, and energy efficiency ratio in broiler production. Eviscerated weight of 58.50% for the treatment group was significantly higher (p<0.05) than 54.10% in the control group. The breast meat yield of Yucca group (32.23%) was significantly higher (p<0.05) than control (30.33%). More frequency of agonistic behavioral expressions was noticed in the control group than the treatment group. A profit of 43.68% was received by usage of Yucca supplementation in the diet on live weight basis. Numerically, lower percentage of moisture was present in Yucca treated group than the control.

**Conclusion:**

From this study, it can be concluded that Yucca supplementation has an important role in augmenting broiler‘s growth performance, efficiency to utilize feed, protein and energy, and survivability. Hence, use of Yucca powder in broiler ration could be beneficial to maintain the litter quality, which directly enhances the productivity in broiler production without any adverse effect.

## Introduction

Broiler industry is one of the most profitable enterprise responsible for employment of rural masses particularly small and marginal farmers. About 66.7% of total output from poultry is coming from broiler sector. The poultry sector has been growing at around 8-10% annually over the last decade with 2.47 million tons of broiler meat [1]. The progress of the poultry industry directly depends on feed costs which represent about 70% of the total production cost [2]. On the other hand, increasing population, changing lifestyle, shifting of food habits, rapid urbanization, increased per capita income, and awareness about health care, etc. are contributing toward rising demand of poultry products. To address the present demand for better productivity and to minimize the feed cost, different non-conventional feeds have been evaluated. *Yucca schidigera* extract is used as a natural medicine, a foaming agent, flavor enhancer in the food and beverage industries, and as an additive for feed in the poultry, swine, and cattle industries. It has also been used successfully to control ammonia accumulation in the animal’s holding facilities, as well as to reduce ammonia concentration and fecal odor in animal excreta [3]. The use of this plant extract in poultry feed enhances metabolic efficiency, control environmental ammonia levels, improve egg weights, feed conversion, and production [4,5]. Saponin, as the main chemical component of *Y. schidigera* extract is present in steroidal form, which physically binds ammonia, reducing the level of free ammonia. *Y. schidigera* had significant effect on the improvement of economic traits, which led to better production and carcass characteristics on broiler chickens [6].

There is a paucity of data regarding usage of this plant extract as poultry feed additives in India. Hence, the present study was planned to evaluate the effect of *Y. schidigera* in broiler performance, carcass quality along with there economic implications.

## Materials and Methods

### Ethical approval

The Institutional Animal Ethical Committee of GADVASU, Ludhiana, Punjab approved the proposed design of the study ensuring that no potential harm toward animal welfare was done and without causing any discomfort to the birds.

### Chicks, treatments and management

Day-old broiler chicks of equal sex ratio were randomly divided into treatment of Yucca supplemented and control group, each having 60 birds in three replications of 20 numbers. The feeding management and rearing conditions were similar for all the groups as per the standard except the Yucca supplementation in the treatment group which was supplemented @ 125 mg/kg of feed. The chicks were protected against New Castle and Infectious Bursal Diseases by routine vaccination. Feed and fresh water were made available *ad-libitum* all the times. The proximate analysis of feeds were done for different contents [7]. The chicks were fed starter diet (2893 KCal ME/kg, 22.01% crude protein [CP]) for first 3 weeks, and the broiler finisher diet (2909 KCal ME/kg, 20.21% CP) for the following 3 weeks (Table-1).

**Table-1:** Ingredient composition of broiler starter and finisher rations.

Ingredient	(Kg/100 kg)	

	Starter	Finisher
Corn, yellow	49.25	55
Soybean meal	41.5	36
Rice polish (oiled)	05	05
Dicalcium phosphate	2.75	02
Limestone powder	01	1.5
Common salt	0.5	0.5
Additives	+	+
Methionine	0.220	0.215

**Calculated chemical composition**		

CP%	22.1	20.21
ME, kcal/kg	2893	2909
Lysine %	1.20	1.18
Methionine %	0.5	0.69
Calcium	1.2	1.24
Available phosphorous	0.5	0.59

Additives included (per 100 kg): Liver tonic (Superlive TM) 0.25 g, vitamin C 20 g, choline chloride 50 g, trace mineral 50 g (Iron 4000 mg, copper 0.5 g, manganese 6000 mg, zinc 4600 mg, selenium 10 mg, iodine 80 mg) vitamin A 825000IU, vitamin D3 165000IU, vitamin E 500 mg, vitamin B12 0.015 mg, vitamin K 100 mg, thiamine 80 mg, riboflavin 6 mg, vitamin B6 160 mg, Niacin 1200 mg, biotin 0.2 mg, folic acid 1.0 mg, TM200 25 g, coccidiostat 50 g. Note: TM200 and coccidiostat were supplied up to 3 weeks of age only. In addition to these supplement, lysine and cysteine were also added to fulfill the requirement. CP=Crude protein

### Growth performance, carcass characteristics, behavior, and litter content

Live weight and feed intake per pen basis were recorded for the calculation of weight gain and feed conversion ratio (FCR) (feed/gain), energy efficiency ratio (EER) (energy intake/gain in live weight), and protein efficiency ratio (PER) (gain in live weight/protein intake) at weekly interval. Record of mortality was maintained on a daily basis. The necropsy examination was done for evaluating any gross pathological lesion and cause of death of each chick. Total mortality in each treatment was then calculated and expressed on a percentage basis. At the end of the experiment, three birds from each replicate were randomly selected from each group and were sacrificed to compare the carcass characteristics [8]. The litter samples were analyzed for different content [7] by proximate analysis in alternate weekly interval. The data pertaining to behaviour of broiler chicks under different groups was recorded using handy cam video recorder (SONY 755E) and the responses of the birds in all the treatment groups were examined [9].

### Economic analysis

The economic viability of *Y. schidigera* supplement for broiler production was evaluated on the basis of total expenditure incurred on the used inputs and the return from the sale of live birds. Being common in both the groups, the general inputs and outputs during the whole study were not considered for economic analysis. Cost of feed was calculated as a sum of the products of the price of different ingredients and their proportionate amount used in the feed. Feed cost was calculated by the average amount of feed consumed in each treatment on phase basis.

### Statistical analysis

The collected data was subjected to statistical analysis using Software Package for Social Sciences (SPSS Version 16.0) available in the Central library, Guru Angad Dev Veterinary and Animal Sciences, Ludhiana. The recorded data was subjected to one-way analysis of variance [10] with comparison among means by Duncan‘s multiple range test [11] with the significance level of p≤0.05.

## Results and Discussion

### Growth performances

**Figure-1:**
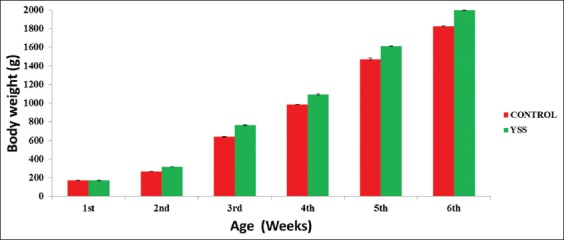
Effect of Yucca treatment on weekly average body weight of broiler chicks in winter season.

The average body weight at 6^th^ week of the experiment was significantly (p<0.05) higher in Yucca group (1995 g) than the control group which lagged behind with the average body weight of 1822 g (Figure-1). Feed intake during the starter phase indicated numerically higher consumption in Yucca group than control group chicks. However, a reversed trend was recorded at finishing stage. The efficiency of utilization of feed was significantly better in Yucca group than control group in both the starter and finisher phase of growth, which led to significantly (p<0.05) better FCR value of 1.91 in Yucca group than that of control (2.10). Overall data for protein efficiency ratio (PER) indicated significant improvement in the efficiency of broiler chicks to convert the protein into body weight gain among the treatment group than those in the control group (Table-2). The values for the EER varied from 5.57 to 6.09 with highest (p<0.05) value in control as compared to Yucca group. During the entire period of experiment, significantly higher protein, energy, and net feed consumption by the chicks of the treatment group associated with more weight gain thus improved FCR, PER, and EER values indicated better efficiency of utilization of feed, protein, and energy in broiler chicks with the Yucca supplementation than control group.

**Table-2:** The production parameters of broiler chicks.

Parameters	Treatments (mean±SE)	

	Control	YSS
Initial average body weight, g	52.10±0.00	52.10±0.00
Final average body weight, g (at 42 days)	1822.16^a^±6.10	1995.17^b^±3.15
Average weight gain, g	1770.06^a^±6.09	1943.08^b^±3.15
Average feed intake, g	3713.80±40.71	3727.00±53.47
FCR	2.10^b^±0.01	1.91^a^±0.03
PER	2.31^a^±0.02	2.52^b^±0.04
EER	6.09^b^±0.05	5.57^a^±0.08
Survivability, %	95.00±2.89	98.33±1.67

Mean value bearing different superscripts in a row differ significantly (p<0.05), SE=Standard error, FCR=Feed conversion ratio, EER=Energy efficiency ratio, PER=Protein efficiency ratio, SE=Standard error

Improvement of final body weight may be related to positive effects of steroid saponins present in Yucca on nutrient absorption from the gastrointestinal tract. Previous studies have demonstrated that steroid saponins can improve the absorption of nutrients by the intestinal tract [12]. The enhanced average weight gain and better FCR under Yucca supplemented group in the experiment conducted during the winter season were attributed to the enrichment of the basal diet with natural saponins from *Y*. *schidigera* which might resulted in emulsification of oil fats, promoting their digestion, and absorption of vitamins and minerals, confirmed to the findings of many researchers [13,14]. However, significant weight gain was observed in the Yucca treated groups while comparing yeast cell walls and *Y. schidigera* extraction in layer hen’s diet [15]. The overall data on growth revealed that the use of Yucca had a significant effect on growth performance, which might be acting as bio-stimulant and growth promoter in broiler chicks. Use of *Y. schidigera* extract also found a better digestibility in rabbit, which might have contributed for growth and production [16].

### Survivability

Lower survivability percentage was recorded in the control group (95%) as compared to the treatment group (98.33%). Similar pattern of mortality [17] with the Yucca supplementation in the layer diet were noticed in the Yucca treated groups while comparing yeast cell walls and *Y. schidigera* extraction in layer hen’s diets.

### Carcass characteristics

Dressed yield and prime cuts of the meat in treatment groups have been shown in Table-3. Eviscerated weight percentage for the treatment group was 58.50% i.e., significantly higher in treatment group (p<0.05) than 54.10% in the control group. The breast meat yield of Yucca group (32.23%) was significantly higher (p<0.05) than control (30.33%). Similarly, thigh yield was also significantly higher in Yucca group than that of the control group. Thus overall yield of edible meat was significantly higher in Yucca group (63.40%) than the control group (59.50%). There was no significant difference observed in drumstick and giblet percentage. The percentage of live weight to breast meat, drumsticks, and thigh indicated that the control groups had lower yield than that of Yucca supplementation group. The significant difference might be due to the mode of application of Yucca in diet. A similar finding on improvement in carcass characteristics was also established [6].

**Table-3:** Effect of different treatment on carcass parameter of broiler chicks.

Parameters	Treatments (mean±SE)	

	Control	YSS
*Eviscerated weight, %	54.10^a^±0.33	58.50^b^±0.79
*Giblet, %	5.53±0.24	4.92±0.33
**Breast, %	30.33^a^±0.53	32.23^b^±0.50
**Thigh, %	16.97^a^±0.33	18.92^b^±0.11
**Drumstick, %	15.8±0.49	15.8±0.42
*Edible, %	59.50^a^±0.55	63.40^b^±0.61
*Inedible, %	40.50^b^±0.64	36.64^a^±0.62

Mean value bearing different superscripts in a row differ significantly (p<0.05),

* Percentage of body weight,

** Percentage of eviscerated weight,

SE=Standard error

### Behavior

The birds under treatment group also had less frequency of agonistic behavioral expressions like pecking as compared to those of the control group. Significantly lower (p<0.05) percentage of birds was observed who avoided other birds in Yucca group than the control group. The control group had higher percentage of both pecking and avoiding than the treatment group. This variation in agonistic behavior of broiler chicks attributed to a strong influence of treatments in relieving the birds from stress and adversity compared to control groups. The increased expression of agonistic behavior by the control birds like pecking, avoiding and more time spent in lying, sitting might be the reason for lethargy, discomfort and stress.

Natural behavioral responses like dust bathing was witnessed with significantly higher percentage in the treatment group as compared to control group birds. Control group spent numerically less time in almost all non-agonistic behavior against the treatment group. The expression of lying, sitting and standing behavior in the control group was significantly (p<0.05) higher than the treatment group. Lower percentage of natural behavior in the control chicks might be due to discomfort from litter containing more moisture content.

### Litter content

The moisture content was not significantly different among the groups throughout the experiment however the moisture content was numerically higher in litter sample of control than that of the treatment group [14]. No specific trend was noticed in the pH and the percent available nitrogen in litter content (Table-4). The increased ash content was recorded in all the groups with the advancement of time with no significant difference in the litter samples within the groups in the entire experimental span.

**Table-4:** Litter quality assessment parameters of various treatment.

Period (week)	Parameters	Treatments (mean±SE)	

		Control	YSS
End of 2^nd^ week	pH	7.3±0.15	7.5±0.06
	Moisture, %	18.67±3.19	16.00±1.53
	N, %	2.10±0.27	2.32±0.25
End of 4^th^ week	pH	6.5±0.17	6.3±0.15
	Moisture, %	20.50±2.84	19.67±2.85
	N, %	4.94±0.51	4.35±0.07
End of 6^th^ week	pH	10.3±0.06	10.2±0.09
	Moisture, %	30.33±1.76	30.00±3.33
	N, %	5.03±0.30	4.74±0.32

Mean value bearing different superscripts in a row differ significantly (p<0.05), SE=Standard error

### Economics

The price of *Y. schidigera* was Rs.1000/kg and the charge for incorporation in feeding was calculated as Rs.4.70 per bird. The feed cost for starter phase was Rs.21.65 and for grower phase was Rs. 20.28 per kg of feed. The total benefit per bird is 57.32% by usage of Yucca supplementation in the diet in comparison to the control was realized. A profit of 43.68% was gained from usage of Yucca supplementation in the diet on live weight basis (Table-5). Economic analysis revealed that the application of these products could be cost-effective management practice to improve shed environment and in turn performance of broiler chicks. The benefits of litter treatment and Yucca supplementation include (1) heavier birds (2) improved feed conversion (3) lower mortality which proves efficiency of Yucca in the improvement of economic traits that in turn develops better economy of production [6].

**Table-5:** Economic impact of treatment on broiler production.

Parameter	Control	YSS
Expenditure		
Total cost of feed consumed per bird in starter phase (Rs)	19.76	20.57
Total cost of feed consumed per bird in finisher phase (Rs)	56.80	56.31
Total feed cost	76.57	76.89
Cost of Yucca powder	0	4.7
Total expenditure	76.57	81.59
Return		
Receipt per bird when sold @ Rs 50 per kg live weight (Rs)	91.11	99.76
Total return	14.54	18.17
Profit (live bird basis)		
Margin of receipt by sale of live birds over feed cost (Rs)	14.54	22.87
Percent difference of margin of receipt from control	0.00	57.32
Profit (unit weight basis)		
Total cost of feed consumed per kg live weight (Rs)	42.02	38.54
Percent difference of feed cost from control per kg live weight	0.00	−8.29
Margin of receipt per kg live weight when sold @ Rs 50 per kg live weight	7.98	11.46
Percent difference of margin of receipt from control per kg live weight	0.00	43.68

## Conclusion

Dietary manipulation with Yucca supplementation @125 mg per kg of feed improved the desirable traits such as weight gain, feed efficiency, and carcass characteristics by improving the micro-climatic conditions and health status of broiler chicks. Hence, Yucca extract could be safely used in broiler rearing for higher economical return without any adversity.

## Authors’ Contributions

SPS, DK, APSS, and AS designed the experiment. SPS carried out the experimental work. DK, APSS, MS, and AS were involved in scientific discussion and analysis of the data. SPS drafted and revised the manuscript. All authors read and approved the final manuscript.
